# Respiratory infections after elexacaftor/tezacaftor/ivacaftor treatment in people with cystic fibrosis: analysis of the European Cystic Fibrosis Society Patient Registry

**DOI:** 10.1183/23120541.01248-2024

**Published:** 2025-08-04

**Authors:** Mordechai Pollak, Simone Gambazza, Annalisa Orenti, Virginia De Rose, Dario Prais, Eitan Kerem, Meir Mei Zahav

**Affiliations:** 1 The Adelson School of Medicine, Ariel University, Ariel, Israel; 2 Pediatric Pulmonology Institute, Schneider's Children's Hospital, Petah Tikvah, Israel; 3 Laboratory of Medical Statistics, Biometry and Epidemiology “G.A. Maccacaro”, Department of Clinical Sciences and Community Health, Dipartimento di Eccellenza 2023-2027, University of Milan, Milan, Italy; 4 Department of Molecular Biotechnology and Health Sciences, University of Turin, Turin, Italy; 5 School of Medicine, Faculty of Medical and Health Sciences, Tel Aviv University, Tel Aviv, Israel; 6 Pediatric Pulmonology Institute, Hadasah Medical Center, Jerusalem, Israel; 7 Faculty of Medicine, The Hebrew University of Jerusalem, Jerusalem, Israel

## Abstract

**Background:**

Elexacaftor/tezacaftor/ivacaftor (ETI) has improved outcomes for people with cystic fibrosis (pwCF). This study evaluated changes in airway microbiological infection status after initiating ETI.

**Methods:**

Using the European Cystic Fibrosis Society registry, pwCF who started ETI between 2019 and 2021 were identified. The changes in microbiological status from 1 year before to 1 year after ETI initiation, were compared with the changes seen from 3 to 1 years before starting ETI. Mixed-effect regression models were used to analyse changes. Data from 2 years after initiation were examined for those starting ETI in 2019–2020.

**Results:**

Included were 15 739 pwCF from 30 countries. In the year before ETI, 38.4% were positive for *Pseudomonas aeruginosa* (PsA) and 36.4% for methicillin-sensitive *Staphylococcus aureus* (MSSA). After ETI, 38.7% of PsA-positive and 47.2% of MSSA-positive patients transitioned to negative status, compared with 14.8% and 29.1%, respectively, in the previous years. The adjusted difference in transitioning to negative was 14.6% (PsA) and 17.1% (MSSA), both p<0.001. Similar improvements were seen for *Burkholderia cepacia* complex and *Stenotrophomonas maltophilia*. For those starting ETI in 2019–2020, PsA positivity remained low over 2 years, decreasing from 46.8% pre-ETI to 30.4% and 27.7% at 1 and 2 years after ETI treatment.

**Conclusion:**

One year after starting ETI, many pwCF who were initially positive for various CF-related pathogens, shifted to a negative status, a change less common before ETI. These findings suggest that ETI reduces airway infections, with benefits extending into the second year of treatment, although some pwCF continue to carry these pathogens despite treatment.

## Shareable abstract

**Many pwCF with initial positivity for CF-related pathogens shifted to a negative status after starting ETI, a shift less common pre-ETI. These findings suggest ETI reduces airway infections, with sustained benefits observed into the second treatment year.** https://bit.ly/4gxC3KU

## Background

Elexacaftor/tezacaftor/ivacaftor (ETI) has revolutionised the management of cystic fibrosis (CF), significantly enhancing the lives of those affected by the condition. ETI, as a highly effective triple-combination therapy, has dramatically improved lung function, reduced the frequency of respiratory infections and enhanced overall quality of life for people with CF (pwCF) [1–6]. By targeting the underlying cause of the disease, by improving the CF transmembrane conductance regulator (CFTR) protein function, ETI has not only led to substantial clinical improvements but also offered hope for a better and more stable life, marking a major advancement in CF care and transforming patient outcomes. However, it is important to note that it does not cure the disease. CF remains a chronic and progressive condition. Patients may still experience some disease-related complications and require ongoing treatment and monitoring to manage their overall health [7–9].

Prior to the introduction of CFTR modulators, bacterial lung infections were known to cause a decline in lung function and shorten survival rates in pwCF [10, 11]. However, the impact of these infections once CFTR function is partially restored remains unclear. Gaining a better understanding of infection rates in this new context could inform clinical decisions, such as the necessity of eradicating specific pathogens. This knowledge is particularly crucial given the ongoing efforts to reduce treatment burdens in the era of highly effective modulator therapy (HEMT) [12, 13]. In this study, we use data of the European Cystic Fibrosis Society Patient Registry (ECFSPR) to assess changes in microbiological status of pwCF following ETI treatment.

## Methods

### The European Cystic Fibrosis Society Patient Registry

The ECFSPR annually collects data on pwCF from CF centres and national CF registries across the World Health Organization's defined region of Europe. The registry contains data spanning from 2008 to 2022. In 2022, data collection included over 50 000 pwCF from 40 countries [14], providing a comprehensive reflection of the CF landscape across the continent. Data are provided to ECFSPR under existing ethical approvals and data-governance structures, and all participants or guardians signed an informed consent prior to data collection. The study was approved by the ECFSPR Scientific Committee and Steering Committee on 6 September 2023, and data used in this analysis were extracted from ECFSPR on 28 November 2023. A detailed description of the ECFSPR can be found online at www.ecfs.eu/ecfspr.

### Study population

The study population included all pwCF with a confirmed diagnosis of CF, who began ETI treatment between 2019 and 2021. Exceptions were made for certain countries: in Belgium, Greece and the Russian Federation, only pwCF starting ETI in 2019 or 2020 were included, as 2022 data had not been validated by the time of data request approval. For Switzerland, only those who initiated ETI in 2019 were included, due to changes in microbiological data collection methods after 2020, which differed from previous years. The pwCF who died during the study period were included until the year of death. ETI was prescribed at the discretion of the treating physician and based on regulatory eligible criteria, local and national reimbursement approvals and compassionate programs.

### Study design

**FIGURE 1:**
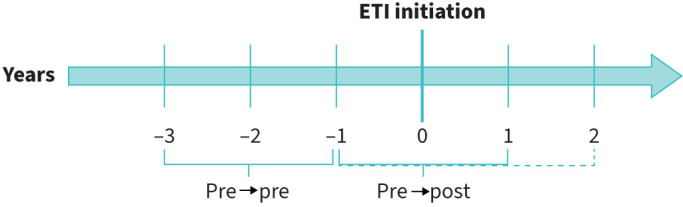
Study timeline. The considered time points for the analysis are shown. “Pre to post” defines the time comprising 1 year before (−1) and after (+1) elexacaftor/tezacaftor/ivacaftor (ETI) initiation (0). “Pre to pre” defines the years before treatment: 3 (−3) and 1 (−1) year before ETI initiation. For those starting ETI in 2019 and 2020 (n=7973) data from the second year after ETI were included.

Changes in the microbiological status of CF pathogens in pwCF were analysed as outlined in figure 1. Microbiological data of pwCF from the year following ETI initiation (+1) were compared with the year prior to ETI initiation (−1). While all pwCF with available data 1 year after ETI initiation were included in the main analysis, many of those who began ETI in 2020 or earlier also had microbiological data available for 2 years post-ETI. For this subset, microbiological status was assessed up to 2 years following ETI initiation. Additionally, we compared the microbiological status of the same individuals in the year prior to initiation (−1) with their status 2 years earlier (−3).

### Microbiological definition

Data on the following pathogens is included in the ECFSPR: Methicillin-sensitive *Staphylococcus aureus* (MSSA), methicillin-resistant *S.* *aureus* (MRSA), *Pseudomonas aeruginosa* (PsA), *Stenotrophomonas maltophilia* (SM), *Burkholderia cepacia* complex (BCC) and *Achromobacter* spp. (AS). Chronic status is defined by ECFSPR when 50% of respiratory samples collected during the last 12 months are positive with at least four samples collected during that period, and/or significantly raised bacteria-specific antibodies according to local laboratories are present [15]. The ECFSPR also labels pwCF microbiological status as chronic when the specified criteria were met in recent years, and there is no indication from the physician that the status has changed. Prior to 2021, PsA, MSSA and BCC were reported annually as chronic/not chronic and SM, MRSA and AS were reported as no/at least once during the year of follow-up. Since 2021, cultures are reported similarly for all pathogens: no/intermittent (defined as at least once but not chronic)/chronic infection. For the present study, the classifications of infections were harmonised along the follow-up years, so that for PsA, MSSA and BCC chronicity was considered, thus chronic status was classified as positive, while intermittent or negative status were classified as negative. On the other hand, for SM, MRSA and AS infection at least one during the year was considered, thus intermittent or chronic status were classified both as positive.

### Statistical analysis

Categorical variables were summarised as number and percentage and by means of graph bars and alluvial plots; numerical variables are shown as mean±sd. Missing data were accounted for and then were excluded when computing descriptive statistics. Linear mixed-effects models were used to evaluate the proportion of pwCF with negative status after ETI among those who were positive before ETI, taking into consideration repeated microbiological status on same pwCF along time points; furthermore, these models were used to test whether the proportion between pwCF who turned negative after ETI was statistically different from any change in proportions observed in the years before ETI; in particular, the analysis looked at the difference in the proportion of pwCF who were negative, among those who were positive between selected time points. Biological sex (female/male), genotype (F508del heterozygote/F508del homozygote/not F508del), percentage of predicted forced expiratory volume in 1 s (FEV_1_ % pred), age at follow-up (years), administration of any CFTR modulator before ETI (yes/no) and gross national income (GNI) per capita in 2022, extracted from the World Bank database (https://databank.worldbank.org/home), were used as adjusting factors. Model estimates are presented as marginal crude and adjusted proportions, with 95% confidence interval (CI). p-values <0.05 were considered statistically significant. Comparison of proportions were assessed using Pearson's chi-squared test. Analyses were conducted with the open-source software R Core Team 2024, v.4.4.0. with the nlme, emmeans and ggalluvial packages added.

## Results

### Population description

A total of 15 739 pwCF aged 26.9±11.6 years (53.1% males) met the inclusion criteria and were included in the analysis (supplementary table S1). Of these, 7973 had 2 years of data available after ETI initiation and were included in the 2-year follow-up analysis. According to the World Bank classification, 4 of the 30 countries included were upper-middle income and the rest were high-income countries. Most patients started ETI in 2020 (48.3%) and 2021 (49.3%). The majority carried at least one F508del mutated allele (43.5% heterozygotes and 54.9% homozygotes), whereas only 241 pwCF (1.5%) were non-F508del. In the year prior to ETI initiation, the mean FEV_1_ % pred was 70.0±24.2%. Treatment with at least one CFTR modulator (*i.e.* ivacaftor, lumacaftor/ivacaftor or tezacaftor/ivacaftor) was reported by 39.1% before starting ETI. Additional demographic and clinical characteristics are shown in table 1.

**TABLE 1:** Characteristics of people with cystic fibrosis (pwCF) at ETI initiation.

	n=15 739
**Age, years**	26.9±11.6 (1.5–86.4)
**Sex, female**	7384 (46.9)
**Genotype**	
F508del heterozygote	6853 (43.5)
F508del homozygote	8645 (54.9)
Not F508del	241 (1.5)
**BMI, kg·m^−2^**	
z-score of children (<18 years)	−0.3±0.98 (−6.16–2.7)
Adults (≥18 years)	22.4±3.4 (12.45–48.6)
**FEV_1_ % pred**	74.0±24.5 (11.9–161.0)
Missing	620
**Pancreatic enzymes**	14 397 (92.7)
Missing	204
**Previous use of CFTR modulators**	
Ivacaftor	609 (4.0)
Missing	390
Lumacaftor/ivacaftor	2955 (19.2)
Missing	362
Tezacaftor/ivacaftor	4209 (27.4)
Missing	367
**Start year of ETI**	
2019	372 (2.3)
2020	7601 (48.3)
2021	7766 (49.3)
***Pseudomonas aeruginosa***	
Positive	5956 (38.6)
Missing	295
**Methicillin-sensitive *Staphylococcus aureus***	
Positive	5461 (35.5)
Missing	358
***Burholderia cepacia* complex**	
Positive	587 (3.8)
Missing	300
**Methicillin-resistant *Staphylococcus aureus***	
Positive	934 (6.0)
Missing	291
***Achromobacter* spp.**	
Positive	1197 (7.7)
Missing	285
***Stenotrophomonas maltophilia***	
Positive	1659 (10.7)
Missing	283

Data are presented as mean±sd (range) or n (%). ETI: elexacaftor/tezacaftor/ivacaftor; BMI: body mass index; FEV_1_ % pred: percentage predicted forced expiratory volume in 1 s; CFTR: CF transmembrane conductance regulator. Age is calculated on 1 January of the follow-up year. BMI is provided as z-scores for children ≥2 and <18 years, otherwise is reported as kg·m^−2^ for pwCF aged ≥18 years. FEV_1_ % pred is reported for pwCF aged ≥6 years.

### Positive microbiological status

**FIGURE 2:**
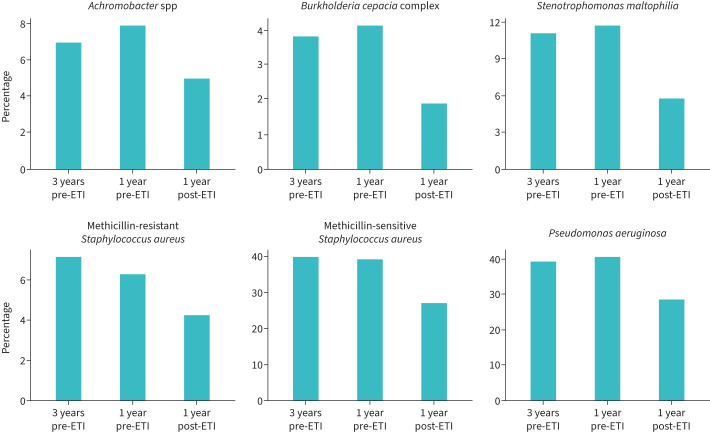
Proportion of positive microbiological status at considered time points. Bars denote the proportion of people with cystic fibrosis with positive microbiological status at considered time points. Specific numbers and percentages for the different time points can be seen in supplementary table S2. ETI: elexacaftor/tezacaftor/ivacaftor.

Overall, 11 245 out of 15 082 pwCF (74.6%) were positive for at least one pathogen in the year prior to ETI. This proportion decreased to 8148 out of 15 158 pwCF (53.8%), 1 year after ETI initiation (p<0.001). In contrast, there was only a minimal change in the number of individuals with at least one positive pathogen 3 years prior to ETI initiation (4552 of 6388, 71.3%) compared with the year before ETI initiation (74.6%) (p<0.001). The proportions of pwCF who were found positive for the specific pathogens at 3 years and 1 year before ETI and 1 year after ETI are shown in figure 2 and supplementary table S2. Overall, the microbiological status of pwCF improved, demonstrating a reduction in positivity rates in the year following ETI initiation. Subsequently, the results of the adjusted model are presented individually for each pathogen, with the magnitude of the coefficients detailed in supplementary table S3.

### Pseudomonas aeruginosa

**FIGURE 3:**
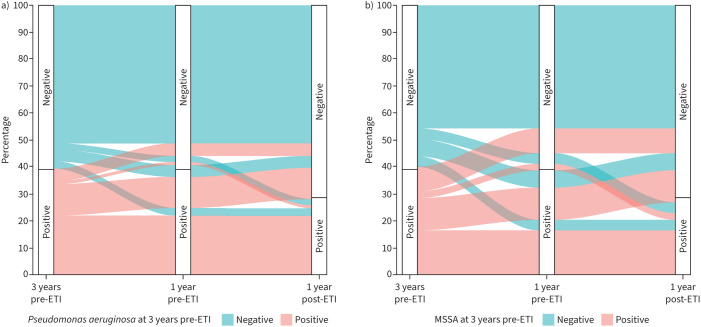
Alluvial plots: *Pseudomonas aeruginosa* status at all time points . Stacked bars denote the percentage of people with cystic fibrosis (pwCF) with a) positive/negative *P. aeruginosa* and b) methicillin-sensitive *Staphylococcus aureus* (MSSA) at different time points. Colours identify pwCF with positive/negative bacterial detection 3 years before elexacaftor/tezacaftor/ivacaftor (ETI). The flow streams show how their frequency changed from 3 years before ETI through 1 year after exposure to ETI.

The distribution and transitions of pwCF across PsA positivity at 3 years before ETI, 1 year before ETI initiation and at 1 year after ETI, are represented in figure 3a. The majority (51.3%) remained negative across years, while 21.8% remained positive. Of those who were PsA positive 3 and 1 year before ETI, 11.6% transitioned to a negative status following ETI. In comparison, 2.3% became positive after starting ETI despite being negative at 3 and 1 years before ETI.

When examining the ETI and pre-ETI periods separately, among those who were PsA positive 3 years before ETI, 14.8% (840 of 5663) changed to negative 1 year before ETI. This corresponds to an estimate of 24.6% (95% CI 19.0–30.3; p<0.001) based on the adjusted model. In contrast, 38.7% (2340 of 6048) of those who were positive at 1 year before ETI became negative 1 year after ETI initiation. This corresponds to an estimate of 39.3% (95% CI 37.5–41.0; p<0.001) from the adjusted model. Overall, the adjusted difference between the two periods (−3 → −1 *versus* −1 → +1) was 14.6% (95% CI 7.3–21.9; p<0.001).

**FIGURE 4:**
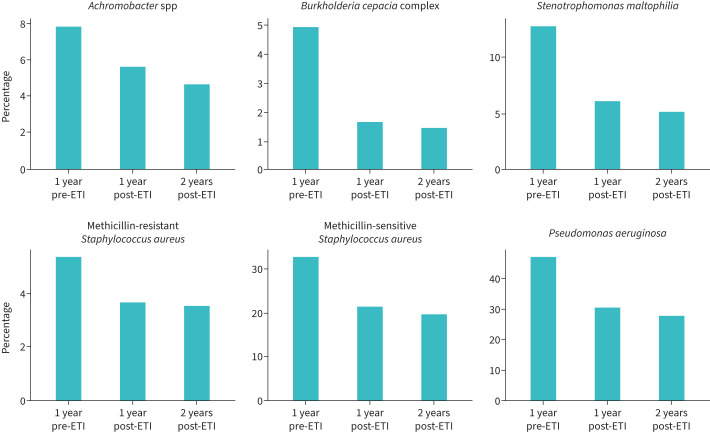
Proportion of positive microbiological status for those starting elexacaftor/tezacaftor/ivacaftor (ETI) in 2019/2020 and with 2 years of follow-up. Bars denote proportion of positive microbiological status for those starting ETI in 2019/2020 with continuous follow-up from −1 to +2 years after ETI. Specific numbers and percentages for the different time points can be seen in supplementary table S4.

To further examine whether this reduction was sustained for a longer period, we assessed PsA positivity in the second year after ETI initiation for those starting ETI in 2019–2020 (figure 4 and supplementary table S4). While 46.8% (3280 of 7016) were PsA positive 1 year before ETI initiation, this proportion decreased to 30.4% (2097 of 6904) 1 year after ETI and further to 27.7% (1901 of 6861) 2 years after ETI.

### Methicillin-sensitive *Staphylococcus aureus*

The proportions of pwCF who were MSSA positive at various time points are shown in figure 3b. The largest proportion (45.7%) remained negative across years, compared with 16.4% who remained positive at all study points.

Of those who were MSSA positive 3 and 1 year before ETI, 12.0% transitioned to negative status following ETI. In comparison, 4.0% became MSSA positive after starting ETI despite being negative 3 and 1 year before ETI

When examining the two periods separately, among those who were MSSA positive 3 years before ETI, 29.1% (1634 of 5611) were negative 1 year before ETI. This corresponds to an estimate of 31.5% (95% CI 27.5–35.6; p<0.001) based on the adjusted model. In contrast, 47.2% (2706 of 5737) of those who were positive before ETI, became negative after ETI initiation, reflected in an estimate of 48.6% (95% CI 46.6–50.5; p<0.001) from the adjusted model. Overall, the adjusted difference between the two periods was 17.1% (95% CI 12–22.1; p<0.001).

For those who started ETI in 2019–2020, 32.8% were MSSA positive 1 year pre-ETI. This proportion decreased to 21.5% 1 year after ETI, and further to 19.6% 2 years after ETI (figure 4 and supplementary table S4).

### *Burkholderia cepacia* complex

An estimated 60.1% (95% CI 59.1–61.1; p<0.001) of those who were BCC positive 1 year before ETI, transitioned to a negative status in the year following ETI initiation. In the years prior to ETI, 17.9% (95% CI 12.6–23.2; p<0.001) transitioned from positive to negative, reflecting a difference of 42.2% (95% CI 35.2–49.2; p<0.001) between the pre-to-post-ETI years. For those who started ETI in 2019–2020, 5% were BCC positive 1 year before ETI. This proportion decreased to 1.6% 1 year after ETI and 1.4% 2 years after ETI initiation (figure 4 and supplementary table S4).

### Methicillin-resistant *Staphylococcus aureus*, *Stenotrophomonas maltophilia* and *Achromobacter* spp.

Since MRSA and AS were reliably documented in the registry only after 2018, there was insufficient data from the pre-ETI period to present valid estimates. However, the frequency of pwCF who were MRSA-positive decreased from 5.4% prior to ETI initiation to 3.7% 1 year after initiation. The proportion of those who were AS positive decreased from 7.8% to 5.6% in the same period.

Among pwCF who were SM positive 3 years before ETI, 802 (50.9%) were negative 1 year before ETI. This corresponds to an estimate 56.6% (95% CI 52.5–60.6; p<0.001) based on the adjusted model. A larger proportion of pwCF who were positive before ETI transitioned to a negative status after ETI (1372, 78.1%), reflected in an estimate of 77.9% (95% CI 76.5–79.4; p<0.001) from the adjusted model. The adjusted difference between the two periods was 21.4% (95% CI 16.0–26.7; p<0.001).

For those starting ETI in 2019–2020, MRSA, SM and AS positivity was reduced 1 year after ETI, with these reductions being sustained at 2 years after ETI (figure 4 and supplementary table S4).

### Pattern of pathogen detection

The pattern of detection of considered pathogens in those who started ETI in 2020, were examined 1 year before, and 1 and 2 years after ETI. Overall, 23.5% were negative for all pathogens at 1 year before ETI, and this proportion was more than doubled in the years after ETI (48.4% and 53.7 at 1 and 2 years after ETI, respectively). A pattern towards lower occurrence of co-infection after ETI can be seen in table 2.

**TABLE 2:** Pattern of the most frequent microbiological combinations in people with cystic fibrosis (pwCF) starting ETI in 2019–2020

Co-detection	1 year pre-ETI (n=7232)	1 year post-ETI (n=7191)	2 years post-ETI (n=7154)
**No detection**	1702 (23.5)	3477 (48.4)	3845 (53.7)
**PsA+MSSA**	768 (10.6)	548 (7.6)	467 (6.5)
**MSSA+SM**	203 (2.8)	75 (1)	57 (0.8)
**PsA+SM**	201 (2.8)	72 (1)	67 (0.9)
**MSSA+AS**	110 (1.5)	72 (1)	62 (0.9)
**PsA+AS**	109 (1.5)	67 (0.9)	65 (0.9)

Data are presented as n (%). ETI: elexacaftor/tezacaftor/ivacaftor; PsA: *Pseudomonas aeruginosa*; MSSA: methicillin-sensitive *Staphylococcus aureus*; SM: *Stenotrophomonas maltophilia*; AS: *Achromobacter* species. The most frequent positive microbiological combinations in pwCF 1 year before ETI and 1 and 2 years after ETI are presented.

## Discussion

This study, using ECFSPR data, shows a significant reduction in PsA, MSSA, BCC, MRSA, AS and SM positivity following ETI initiation. To our knowledge, this is the first study to show real-world microbiological data following ETI in a large international cohort, including a diverse population.

It has been previously shown that HEMT influences lung infection in pwCF: ivacaftor, the first approved modulator was linked to a reduced occurrence of sputum cultures testing positive for PsA [16–18]. Regarding ETI, one study showed sputum densities of common CF pathogens significantly decreased 1 month after initiation, but in most cases the pathogens were not totally eradicated 6 months after ETI initiation [19]. Another, relatively small, single-centre study showed a significant reduction in PsA colonisation after 2 years of ETI use [20]. Recently, a local German CF registry-based study showed a reduction in MSSA-positive cultures from 54.3% to 44.3% and 40.2% and in PsA from 39.9% to 31.9% and 22.6% at 3 and 21 months, respectively. This reduction was associated with age, previous lung function and pre-ETI colonisation status [21]. In this current study, we detected a significant reduction in the positivity of all assessed pathogens up to 2 years after ETI initiation.

Along with the significant reduction in detection of specific pathogens, we also found that the proportion of pwCF with negative status for all assessed pathogens, more than doubled over 2 years following ETI initiation. It is well recognised that pwCF using HEMT expectorate significantly less sputum, which may reduce the amount of collected samples. Using the current methodology, the extent of this reduction could not be estimated, as ECFSPR reports only annual positive culture results without specifying the number of cultures obtained. However, monitoring airway pathogens and antibiotic treatments has been, and still is an important practice in pwCF [22]. Current guidelines continue to advocate for routine sputum collection, among other ways, by encouraging induced sputum techniques. It is reasonable to assume that most centres adhere to these guidelines, but sputum collection rates are not documented by ECFSPR and therefore this could not be confirmed. Additionally, the ECFSPR states that a subject previously chronically infected should be reported as non-chronic only if there is clinical evidence supporting this change. Therefore, in cases where no sputum was collected over the year, patients can still be reported as chronically infected. The ECFSPR guidelines of reporting positive airway infection, based on previous years, even when there are no cultures available, might overestimate the rate of bacterial infection in the HEMT era when obtaining cultures is more difficult.

So far, practices for PsA eradication or maintenance antibiotic therapy for PsA have remained unchanged [23]. In the ETI era, efforts are made to reduce the burden of CF care in a safe way [12, 13]. While this study did not focus on the clinical benefits for those that reduced the detection of CF-related pathogens, the fact that much less patients are reported as chronically infected with PsA after ETI, can encourage clinicians to insist on proper culture sampling, and when appropriate, to carefully explore options for reduction in antibiotic treatments.

Despite the overall significant reduction in pathogen detection following ETI initiation, a small proportion of pwCF experienced the opposite trend. Specifically, 2.3% and 4% of individuals who were PsA or MSSA negative, respectively, shifted to positive status for these pathogens after starting ETI. This was significantly less than the numbers seen in the years prior to ETI (11.9% and 17.2% for PsA and MSSA, respectively). These encouraging results suggest that ETI not only reduced pathogen detection in individuals who had already acquired them but also slowed the natural progression of acquiring new airway pathogens over time. Despite that, the proportion of pathogens acquired after starting ETI is not negligible. Especially for individuals who began ETI after significant and irreversible lung damage had already occurred, managing lung infections is likely to remain a lifelong challenge.

The main strength of this study is the large and diverse population included in the study. Data included in the ECFSPR are collected and reported annually, therefore reducing biases that are often subject in retrospective analyses studies and presenting real-world data. However, the present study has some limitations that should be considered. The microbiological definitions used by the ECFSPR have evolved over the years and were slightly different for different pathogens. We therefore had to harmonise definitions to have comparable data. Also, in middle- and high-income countries, most pwCF who qualify for ETI therapy started treatment between 2019 and 2021. Therefore, a comparator group with similar characteristics, in terms of socioeconomic levels and CFTR mutations but without ETI treatment, was not available. Since pwCF with stop mutations [24] and those from lower GNI countries [25] typically have a poorer prognosis, they are less suitable for use as comparator groups. Instead, to isolate the effect of ETI on pathogen detection, we used data from the same patients, over 2 years prior to ETI as a proper comparison. Hence, other factors that change over time could potentially confound our results. For example, the prevalence of airway pathogens in pwCF often changes with age [26]. Notably, PsA infections usually increase with time, so the observed reduction in PsA after ETI, despite the passage of time, underscores the significance of our results.

Another potential confounding to our results could be the impact of the COVID-19 pandemic, which led to fewer in-person visits and possibly fewer cultures being obtained. Additionally, isolation practices and mask-wearing may have influenced infection rates. While it is difficult to quantify the exact effect of the pandemic, the limited reduction in positivity during the pandemic years before ETI initiation, coupled with the sustained reduction observed after ETI initiation, even after the peak of the pandemic had passed, suggests that the pandemic's impact on our findings was limited.

In conclusion, 1 year after ETI initiation, a significant proportion of pwCF with a positive microbiological status transitioned to negative. This proportion was notably lower before ETI initiation, suggesting that this change is likely attributable to ETI therapy. Additionally, the reduction seems to be sustained into the second year after ETI initiation. The clinical significance of these findings warrants further investigation in future studies.

## Supplementary material

**Please note:** supplementary material is not edited by the Editorial Office, and is uploaded as it has been supplied by the author. Supplementary material 01248-2024.SUPPLEMENT
