# Supplementary material for: Respiratory infections after elexacaftor/tezacaftor/ivacaftor treatment in people with cystic fibrosis: analysis of the European Cystic Fibrosis Society Patient Registry
Source: ERJ Open Res. 2025 Aug 4;11(4):01248-2024. doi: 10.1183/23120541.01248-2024 (PMC12320108; doi:10.1183/23120541.01248-2024)
Supplement: Supplementary file 1 [file 01248-2024.SUPPLEMENT.pdf]

**Supplement**:

Supplementary table S1- countries of origin

| | N = 15,739 |
|---|---|
| Country | |
| Austria | 234 (1.5%) |
| Belgium | 109 (0.7%) |
| Bulgaria | 7 (0.04%) |
| Croatia | 15 (0.1%) |
| Cyprus | 6 (0.04%) |
| Czech Republic | 240 (1.5%) |
| Denmark | 333 (2.1%) |
| France | 2,741 (17.4%) |
| Germany | 3,209 (20.4%) |
| Greece | 38 (0.2%) |
| Hungary | 4 (0.03%) |
| Iceland | 6 (0.0%) |
| Ireland | 657 (4.2%) |
| Israel | 213 (1.4%) |

| | N = 15,739 |
|---|---|
| Italy | 1,347 (8.6%) |
| Luxembourg | 1 (0.01%) |
| Netherlands | 126 (0.8%) |
| Norway | 2 (0.01%) |
| Poland | 50 (0.3%) |
| Portugal | 89 (0.6%) |
| Romania | 1 (0.01%) |
| Russian Federation | 7 (0.04%) |
| Serbia | 6 (0.04%) |
| Slovak Republic | 36 (0.2%) |
| Slovenia | 45 (0.3%) |
| Spain | 287 (1.8%) |
| Sweden | 49 (0.3%) |
| Switzerland | 22 (0.1%) |
| Turkey | 43 (0.3%) |
| United Kingdom | 5,816 (37.0%) |

Data are presented as counts and percentages (%).

Supplementary Table S2 – positive microbiological status at considered time points

| | Timepoints | | |
|---|---|---|---|
| **Pathogens** | **3 yrs pre-ETI** | **1yr pre-ETI** | **1yr post-ETI** |
| AS | 446/6477(6.9%) | 1198/15261(7.9%) | 754/15229(5%) |
| BCC | 559/14691(3.8%) | 634/15354(4.1%) | 286/15238(1.9%) |
| SM | 1609/14553(11.1%) | 1791/15343(11.7%) | 859/15231(5.6%) |
| MRSA | 461/6485(7.1%) | 954/15266(6.2%) | 640/15219(4.2%) |
| MSSA | 5692/14315(39.8%) | 5907/15248(38.7%) | 4095/15220(26.9%) |
| PsA | 5776/14694(39.3%) | 6244/15345(40.7%) | 4348/15238(28.5%) |

*Numbers are absolute frequencies and percentages (%)*

Supplementary Table S3 – Magnitude of coefficients in the adjusted models for each pathogen.

| **Adjusting factors** | **PsA*** | **MSSA** | **MRSA** | **BURKHO** | **MALTO** | **ACHRO** |
|---|---|---|---|---|---|---|
| **Sex (*females vs males*)** | -3.9 95%CI(-5 to -2.8)%<br>p-value<0.001 | 0.9 95%CI(-0.3 to 2.1)%<br>p-value=0.159 | 0.2 95%CI(-0.4 to 0.7)%<br>p-value=0.588 | -0.1 95%CI(-0.4 to 0.2)%<br>p-value=0.572 | -1.3 95%CI(-2 to -0.6)%<br>p-value<0.001 | -0.8 95%CI(-1.4 to -0.3)%<br>p-value=0.005 |
| **Age (yrs)** | -0.2 95%CI(-0.2 to -0.1)%<br>p-value<0.001 | 0.2 95%CI(0.1 to 0.2)%<br>p-value<0.001 | 0.03 95%CI(0 to 0.1)%<br>p-value=0.043 | 0 95%CI(-0.02 to 0.02)%<br>p-value=0.965 | 0 95%CI(-0.03 to 0.04)%<br>p-value=0.974 | 0 95%CI(-0.02 to 0.03)%<br>p-value=0.808 |
| **F508del Homozygotes *vs* F508del heterozygotes** | 3.7 95%CI(2.5 to 5)%<br>p-value<0.001 | 3.3 95%CI(1.9 to 4.8)%<br>p-value<0.001 | 0.8 95%CI(0.2 to 1.4)%<br>p-value=0.013 | 0.3 95%CI(-0.1 to 0.7)%<br>p-value=0.102 | 1.1 95%CI(0.3 to 2)%<br>p-value=0.007 | 0.5 95%CI(-0.1 to 1.2)%<br>p-value=0.121 |
| **Not F508del *vs* F508del heterozygotes** | -4.4 95%CI(-9 to 0.1)%<br>p-value=0.058 | 7 95%CI(1.9 to 12.2)%<br>p-value=0.008 | -3.3 95%CI(-5.6 to -1)%<br>p-value=0.005 | 1.3 95%CI(-0.1 to 2.8)%<br>p-value=0.076 | 0.8 95%CI(-2.2 to 3.9)%<br>p-value=0.594 | 0.3 95%CI(-2 to 2.7)%<br>p-value=0.774 |
| **ppFEV$_1$** | 0.2 95%CI(0.1 to 0.2)%<br>p-value<0.001 | -0.03 95%CI(-0.1 to 0)%<br>p-value=0.064 | 0.02 95%CI(0.01 to 0.04)%<br>p-value=0.001 | 0.01 95%CI(0 to 0.02)%<br>p-value=0.006 | 0.02 95%CI(0 to 0.03)%<br>p-value=0.057 | 0.04 95%CI(0.03 to 0.06)%<br>p-value<0.001 |
| **GNI** | -0.2 95%CI(-0.7 to 0.4)%<br>p-value=0.51 | -0.7 95%CI(-1.3 to -0.1)%<br>p-value=0.03 | 0.3 95%CI(0.04 to 0.6)%<br>p-value=0.024 | 0.1 95%CI(-0.1 to 0.3)%<br>p-value=0.291 | -0.3 95%CI(-0.6 to 0.1)%<br>p-value=0.14 | -0.1 95%CI(-0.4 to 0.2)%<br>p-value=0.414 |
| **CFTR modulator before ETI** | -2.1 95%CI(-3.4 to -0.7)%<br>p-value=0.002 | -2.3 95%CI(-3.8 to -0.8)%<br>p-value=0.002 | -0.2 95%CI(-0.9 to 0.4)%<br>p-value=0.476 | -0.1 95%CI(-0.5 to 0.3)%<br>p-value=0.673 | -0.5 95%CI(-1.4 to 0.3)%<br>p-value=0.217 | -0.2 95%CI(-0.9 to 0.5)%<br>p-value=0.579 |

*Numbers are percentage with 95% Confidence Intervals and p-values. * E.g., Females are less Pa negative post ETI than males, nearly 4% less.*

Supplementary Table S4 – positive microbiological status for those starting ETI in 2019/2020 and with two years of follow-up

| | Timepoints | | |
|---|---|---|---|
| **Pathogens** | **1 yr pre-ETI** | **1yr post-ETI** | **2yrs post-ETI** |
| AS | 552/7035(7.8%) | 387/6904(5.6%) | 320/6846(4.7%) |
| BCC | 348/7020(5%) | 113/6906(1.6%) | 99/6864(1.4%) |
| SM | 899/7036(12.8%) | 418/6901(6.1%) | 353/6865(5.1%) |
| MRSA | 377/7037(5.4%) | 252/6903(3.7%) | 242/6856(3.5%) |
| MSSA | 2299/7018(32.8%) | 1487/6904(21.5%) | 1348/6862(19.6%) |
| PsA | 3280/7016(46.8%) | 2097/6904(30.4%) | 1901/6861(27.7%) |

*Numbers are absolute frequencies and percentages (%)*
